# The two-region multi-depot pickup and delivery problem

**DOI:** 10.1007/s00291-018-0534-2

**Published:** 2018-10-16

**Authors:** Adria Soriano, Margaretha Gansterer, Richard F. Hartl

**Affiliations:** Department for Business Administration, University of Vienna, Oskar-Morgenstern-Platz 1, 1090 Vienna, Austria

**Keywords:** Pickup and delivery, Multi-modal transportation, Adaptive large neighborhood search

## Abstract

Logistics networks are constantly evolving such that new and more varied structures arise and need to be studied. Carriers are aiming for opportunities to save costs by efficient planning. Motivated by this, we define the two-region multi-depot pickup and delivery problem. A region in this setting refers to an area where customers and depots are located. We differentiate two kinds of requests depending on whether their customers are located in the same region or not. Due to geographical characteristics, direct transportation between different regions is considered inefficient and a long-distance transportation mode needs to be used to connect them. Hence, we face a complex problem where interrelated decisions are to be made. We propose a decomposition into three subproblems, which relate to well-known problems in the literature. For solving the global problem, an adaptive large neighborhood search (ALNS) algorithm is developed. The algorithm mixes operators tailored to each of the different decisions of each subproblem. We demonstrate that these operators are efficient when applied to problems of their primal nature. In an extensive computational study, we show that the proposed ALNS dominates alternative ALNS schemes, where subproblems are treated sequentially. A detailed analysis of the solution convergence is provided. The proposed approach is a powerful tool to tackle complex decision problems in large distribution networks.

## Introduction

In the last decades, logistics service providers have experienced huge changes. The role of final customers has switched from a passive position to an active one, where the ability to satisfy their needs and wishes regarding time and costs is a capital element for competition between companies. This, together with the continuously increasing quantity of parcels in the market, has put a lot of pressure on carriers. It results in the need to better control operational costs while, at the same time, making infrastructural investments necessary to remain competitive (Allen et al. 2017). Consequently, transportation companies are continuously looking for ways of improving efficiency in their operations. This has led to extensive research in the transportation field during the last years. However, most of the literature has focused on simple transportation network configurations, although many variants with complex restrictions have been studied in an attempt to shorten the gap between theoretical models and reality. These network configurations are normally one-level decision problems, generally consisting of last-mile routing or hub location problems. The increasing importance of transportation in logistics requires to look at more complex problem structures, integrating different operational levels within a supply chain.

Nowadays, many situations can be found where a high volume of transportation requests have to be serviced on a daily basis between distant areas or regions, like different cities or countries. These requests consist mainly on picking up some goods in one area and delivering them in another area. The service of pickup or delivery customers within a region is usually done with light-duty trucks (LDTs). However, the distance and volume of goods to be transported between two regions make the use of these vehicles for inter-region transport quite inefficient. Therefore, other means of transport operating between the regions are needed. In some cases, like in national post services, where many regions have to be serviced, a network of hubs and depots in different levels is set in a way that a maximum coverage is guaranteed. The transportation between two distant regions is hence carried out through the different levels, so no direct transportation exists. Nevertheless, in other cases where less regions are served, setting this kind of network is not efficient. For example a company having production plants in two different regions and needing products or resources to be continuously transferred from one site to the other. This setting might also be relevant for a group of small-sized transportation companies, who decide to merge in a bigger company or to form a coalition in order to be competitive on the long distance against the big firms (see Gansterer and Hartl 2018). These companies would need to find an efficient way of servicing their requests by aggregating and consolidating goods in their depots in one region, and sending them to their depots in other regions. For this type of transportation, typically heavy-duty vehicles (HDVs) are operated.

In line with all described above, this paper works on the concept of multiple regions in pickup and delivery problems (PDP). These problems consist in two or more geographically separated areas, with at least one depot in each of them. A set of pickup and delivery requests, whose origins and destinations are located in different regions, have to be serviced. Normally, other requests with endpoints in the same region can be part of the problem, too. These last requests would be of the classical type found in most PDP literature. In the remainder of this paper, these requests will be referred to as inter-region and intra-region requests, respectively. A customer is considered any service point in a region being either a pickup or a delivery point. Customers are visited by LDTs performing tours on an intra-region basis. Goods belonging to an inter-region request are first consolidated in a depot in the pickup region and then transported to the depot in the destination region. Therefore, the problem setting requires the use of two modes of transportation: transportation within a region (intra-region) and between regions (inter-region). For a better readability, both modes will be referred as short-haul and long-haul transportations, respectively. Long-haul vehicles can be HDVs, but also as other long-distance means of transport like rail or air transportation. The reader interested in multi-modal freight transportation planning is guided to a survey presented in SteadieSeifi et al. (2014).

**Fig. 1:**
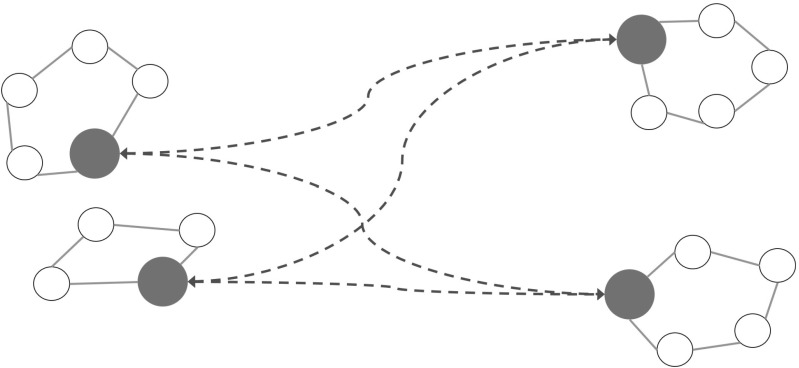
Possible structure of a network for the 2R-MDPDP with 2 depots in each region

To the best of our knowledge, there is no previous work addressing these specific settings on a transportation problem. Considering the above-mentioned relevance of these kind of problems for nowadays logistics, we present the two-region multi-depot pickup and delivery problem (2R-MDPDP) in multiple periods. This problem is a variant of the general setting of the family of multiple regions problems described above. It is composed of two regions and two or more depots per region. For some possible applications like the aforementioned carrier collaborations, situations with two regions seem applicable due to the quantity of carriers working in overlapping areas. In our study, multiple periods are considered. All requests have therefore due dates for performing their services. Further details of the problem are given in Sect. 3. Figure 1 shows a graphical representation of a possible structure of the problem’s network.

The 2R-MDPDP is a two-level transportation problem where two different main decisions are to be made. On the first level is the scheduling decision, where inter-region requests have to be assigned to HDVs. These vehicles travel from the depot where they are placed to another depot in the other region. On the second level is the routing decision, where customers (being either pickup or delivery points) have to be visited by an LDT. This vehicle starts and ends at the same depot and must respect depot operation times. The depot operates in the same region where the customer is located. These two decisions are not independent from each other: for each inter-region request, the depots from where it is serviced by a LDT are the same depots used by the HDV to transport goods between the regions.

The presented study has several important contributions. Firstly, we present the 2R-MDPDP in a multiple period setting, together with a mixed integer programming (MIP) formulation. We find a decomposition of the problem into three subproblems, which relate to well-known problems in the literature. An adaptive large neighborhood search (ALNS) algorithm is devised for solving the problem. The algorithm mixes operators of different nature to deal with the different kinds of elements and decisions related to each subproblem. These operators prove efficient when applied to problems of their primal nature. Finally, in an extensive computational study, we show that the proposed framework dominates alternative ALNS approaches, where the subproblems are treated sequentially.

The remainder of the paper is organized as follows. Section 2 gives an account of the related work existing in the literature. In Sect. 3, the problem is described in detail and a MIP formulation is given. All algorithms used for the computational experiments are explained in Sect. 4. Section 5 presents the computational results. Finally, conclusions are summed up in Sect. 6.

## Literature review

PDPs in multiple regions have received very little attention in the literature so far. To the best of our knowledge, they are firstly discussed in a recent work presented by Dragomir et al. (2018). The authors discuss the relevance of multiple regions logistic problems and present a mathematical model. However, no efficient solution methods have been presented so far.

Literature on single-region vehicle routing problems with pickup and deliveries (VRPDP) is, however, quite extensive. Savelsbergh and Sol (1995) define the general routing problem (GDP) as a vehicle routing problem where pickup and delivery customers have to be served. They describe the PDP as a special case of GDP problems. PDPs are defined in Cordeau et al. (2008) as routing problems where a set of vehicles, starting and ending in a depot, must satisfy a set of requests. A request in this case consists of a pair of pickup and delivery locations between which goods have to be transported. For further information on VRPDPs, we refer the reader to the surveys of Parragh et al. (2008) and Berbeglia et al. (2007). Both of these works make an extensive analysis on all problem variants for VRPDPs existing in the literature. However, each of them proposes a different classification and nomenclature for them.

Most of the aforementioned studies assume a single depot setting. Although multiple depots are of high relevance in real-world settings (see Sect. 1). Nagy and Salhi (2005) consider the problem of VRPDP with mixed backhauls (defined as VRPMB in Parragh et al. 2008) from both a single and multi-depot perspective. This problem is a special case of the VRP where pickup and delivery customers do not need to be paired. The authors propose a heuristic for the single depot problem, based on the application of different routines over an initial solution and present an adaptation to the multi-depot case. Min et al. (1992) also address a problem akin to the VRPMB, but in this case all delivery customers must be served before the pickups. They consider the multi-depot scenario and propose a three phase algorithm for solving it. In their method, the clustering and assignment of customers to depots and routes is performed prior to the route optimization step. A multi-depot heterogeneous PDP with soft time windows is presented by Bettinelli et al. (2014). Note that all these studies consider a single region.

In our study, we assume that location decisions are fixed. If this is not the case, the additional complexity of location-routing problems (LRP) arise. This problem class has been extensively discussed in the literature. In a very recent work, Capelle et al. (2019) propose an exact method for the location-routing problem with pickup and delivery. An LRP with inter-hub transport and multi-commodity pickup and delivery is investigated by Rieck et al. (2014). Crevier et al. (2007) solve an extension of the multi-depot VRP in which vehicles may be replenished at intermediate depots along their route. Calvete et al. (2016) present a two-stage transportation problem in which they integrate elements from both two-echelon problems and LRP. They optimize the flows from hubs to depots and from depots to customers, while deciding which depots to open. However, this work does not cover the routing-level decisions. Reviews on location-routing problems are provided by Laporte and Nobert (1987), Prodhon and Prins (2014), and Drexl and Schneider (2015).

Some similarities with the 2R-MDPDP can be found in the studies by Ghilas et al. (2016a, b). They investigate the utilization of public transport lines for connecting pickup and delivery customers and service them through LDT from the end points of the public line. However, this work considers a single period problem and scheduled departure times for the vehicles. Also Zäpfel and Wasner (2002) and Wasner and Zäpfel (2004) do some related work on the design of a hub and spoke network for a real case of an Austrian parcel provider. They highlight the importance of integrating the long-haul and short-haul transportation in Zäpfel and Wasner (2002) and incorporate the decision of opening new hubs to a similar problem in Wasner and Zäpfel (2004).

With respect to the general structure of the problem, some similarities exist between the 2R-MDPDP and the families of multi-level transportation problems, especially with multi-echelon vehicle routing problems and location-routing problems. The family of multi-echelon vehicle routing problems deal with multiple transportation levels. Goods start at a hub in level one and are transported to a satellite in each level by level-dependent vehicles, until they reach their destination via last-mile routes.

As with the 2R-MDPDP, all transportation decisions are dependent on the others, and an integration of all decisions is needed on the search for optimal solutions. For more details on this problem, we refer the interested reader to Perboli et al. (2011), Crainic et al. (2011) and Breunig et al. (2016) for two-echelon problems, and to Dondo et al. (2011) for the multi-echelon version. A typology of multi-depot PDPs in multiple regions is provided in Dragomir et al. (2018).

## Problem definition and mathematical formulation

The main characteristics of the 2R-MDPDP are inherited from the description of multiple region pickup and delivery problems given in Sect. 1. That is, we have two independent regions, several depots in each region, two modes of transportation (LDT and HDV), and a set of pickup and delivery requests to be transported. The problem is considered for multiple days. All requests have an earliest day of pickup and a latest delivery day. These days represent when the goods will be ready for pickup, as well as when these goods are allowed to be delivered at the latest.

**Fig. 2:**
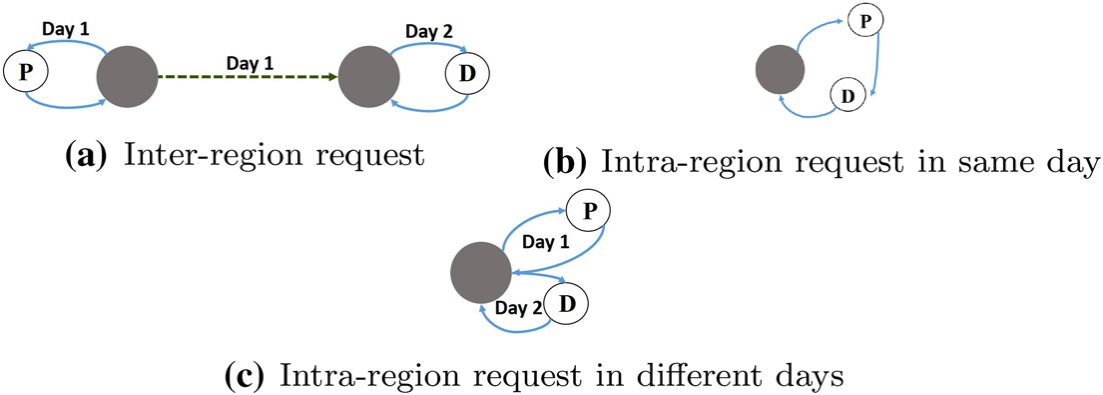
Service options for inter-region and intra-region requests. P and D nodes, respectively, correspond to the pickup and delivery locations of the depicted request type

The problem at hand is composed of two kinds of pickup and delivery requests, inter-region and intra-region requests. On the one hand, inter-region requests have their customers located in different regions, thus needing to be transported from one region to the other with an HDV. Servicing an inter-region request means: (1) picking up the goods at its pickup point with an LDT in region A (if pickup point is in region A, B otherwise), (2) consolidating goods at the depot corresponding to the vehicle and sending them with an inter-region vehicle to region B (region A otherwise), (3) consolidate goods in the destination depot of region B (region A otherwise) from where goods will be dispatched, and deliver them with an LDT. On the other hand, intra-region request customers belong to the same region, so the request is serviced exclusively in short-haul routes. It must be noticed that an intra-region request belong to one of the regions and can only be served from a depot located in that region. Servicing an inter-region request means: (1) picking up the goods at its pickup point with an LDT in region A (if pickup point is in region A, B otherwise), (2) consolidating goods at the depot corresponding to the vehicle and sending them with an HDV to region B (region A otherwise), (3) consolidate goods in the destination depot of region B (region A otherwise) from where goods will be dispatched, and deliver them with an LDT. For intra-region requests, goods do not have to travel between regions. Classical pickup and delivery problems in a single region often force pickup and delivery customers to be part of the same route. This can have some negative effects in the cases where the customers lie in opposite directions with respect to the depot. Hence, we consider the possibility for goods to be stored in the depot and to be delivered in posterior days after the pickup date. In this case, either they are visited on the same day (hence belonging to the same route) or the goods are sent to the depot and delivered in later periods. Consequently, when visited on the same day, precedence constraints must hold for both customers of an intra-region request. Furthermore, we assume that HDVs do not necessarily have to go back to their point of departure. Figure 2 illustrates servicing options for inter-region and intra-region requests. In any case, each request must be served between its earliest and latest service day.

The optimization goal of the 2R-MDPDP consists of minimizing the costs of transporting all inter-region and intra-region requests within their service due days. Costs related to the routing of vehicles are assumed directly proportional to distance. Additionally, we include a fixed cost for the utilization of an LDT on a given day. With this, we pretend to also minimize the number of short-haul routes performed each day, as it is common in VRP problems. The cost for the use of an HDV is proportional to the distance between the starting and ending depot of the vehicle. Maximum transportation capacities are imposed both for LDTs and HDVs. For solving this problem, some assumptions are made: (i) long-haul transportation is assumed to happen at any time after it is ready to be performed, that is, after all the goods assigned to the HDV have arrived to the depot with the short-haul routes. However, goods transported from one region to the other are not assumed to be ready for delivery until the beginning of the following day after long-haul transportation. That means that no inter-region request can be completely served during the same day, (ii) no limitation on the number of available vehicles of both types in each depot is considered.

With the 2R-MDPDP, we are facing an NP-hard problem, since it can be decomposed into three different optimization problems that belong to the class of NP-hard problems by themselves. On the first level, we are facing a scheduling problem, where we need to solve the allocation of requests to HDVs on a multiple period setting. It is constrained by the capacity of the HDV and the service days of each inter-region request. On the second level, we have to solve a routing problem where customers can be paired or unpaired pickup and delivery nodes. In this sense, we are facing on the one hand a multi-depot PDP, since intra-region requests can be assigned to any depot in the same region. On the other hand, we face a multi-period VRPMB problem for each depot (see Sect. 2). If the problem does not contain any intra-region request, we face a scheduling problem coupled with a multi-period VRPMB. If, contrarily, there are no inter-region request, we are left with a multi-depot multi-period PDP (see Table 1).

**Table 1:** Subproblem type classification

		Inter-region request	
		Yes	No
Intra-region request	Yes	Scheduling $+$ VRPMB $+$ multi-depot PDP	Multi-depot PDP
	No	Scheduling $+$ VRPMB	

We propose a mathematical formulation based on the paper from Dragomir et al. (2018), with the addition of a multiple period setting. For simplicity in the notation, copies of depot *j* representing starting and ending depot, respectively, in each region *u* will be denoted as $j_{0}^{u}$ and $j_{1}^{u}$. Besides, ${\bar{u}}$ will be used to denote the region different from region u ($U\setminus u$).

The following **sets** are used:

- *U* set of regions = $\{1, 2\}$
- $N^{u}$ set of inter-region requests starting in region $u = \{1,\ldots ,n\}$
- $R^{u}$ set of intra-region requests served in region $u = \{1,\ldots ,n\}$
- $P^{u}$ depots in region *u*
- $V^{u}$ set of nodes to visit in each region *u*, including depots
- $K_{j}^{u}$ set of LDTs in depot *j* in region *u*
- $$K^{u}$$ $$\cup _{j \in L^{u}}K_{j}^{u}$$
- $L_{j}^{u}$ set of HDVs in depot *j* from region *u*
- *T* set of days

The needed **parameters** are:

- $c_{ii'}^{u}$ travel cost between points *i* and $i'$ in region *u*
- $d_{jj'}^{u}$ travel cost between depots $j\in P^{u}$ and $j' \in P^{{\bar{u}}}$
- $\varGamma$ fixed cost for using a LDT
- $D_{i}$ demand of request *i*
- $q_{i}^{u}$ quantity load on customer *i* in region $$u {\left\{ \begin{array}{ll} D_{i}, \text {if it is a pick up point}\\ -\,D_{i}, \text { if it is a delivery point} \end{array}\right. }$$
- $E_{i}$ earliest pickup day for request *i*
- $A_{i}$ latest delivery day of request *i*
- $Q_\mathrm{SH}$ load limitation for LDTs
- $Q_\mathrm{LH}$ load limitation for HDVs

**Decision variables** are defined as follows:

- $$X_{ii'k}^{ut}$$ $${\left\{ \begin{array}{ll} 1, \hbox { arc }i-i'\hbox { in region }u\hbox { covered by vehicle }k \in K^{u} \hbox { on day }t, i,i' \in V^{u} \\ 0, \text { otherwise} \end{array}\right. }$$
- $$Y_{ijj'l}^{ut}$$ $${\left\{ \begin{array}{ll} 1,\hbox { request }i \hbox { transported in HDV }l \hbox { on period }t, j\in P^{u}, j'\in P^{{\bar{u}}}\\ 0, \text { otherwise} \end{array}\right. }$$
- $$Z_{k}^{ut}$$ $${\left\{ \begin{array}{ll} 1,\hbox { if LDT }k \in K^{u}\hbox { is used on day }t\\ 0, \text { otherwise} \end{array}\right. }$$
- $$O_{jj'l}^{ut}$$ $${\left\{ \begin{array}{ll} 1, \hbox { HDV }l\hbox { from }j \in P^{u} \hbox { to }j' \in P^{{\bar{u}}} \hbox { used on period }t\\ 0, \text {otherwise}\end{array}\right. }$$
- $$Q_{ik}^{ut}$$ $$\hbox { Quantity loaded after visiting node } i \in V^{u}\hbox { in vehicle }k \in K^{u}\hbox { on period } t$$
- $$S_{ik}^{ut}$$ $$\hbox { Time of service in node }i \in V^{u}\hbox { by vehicle }k\hbox { on period }t$$

1 $$\begin{aligned} \begin{aligned} Min&\sum _{u \in U}\sum _{i,i' \in V^{u}}\sum _{k \in K^{u}}\sum _{t\in T}c_{ii'}^{u}\cdot X_{ii'k}^{ut} +\sum _{u \in U}\sum _{j \in P^{u}}\sum _{j' \in P^{{\bar{u}}}}\sum _{l \in L^{u}}\sum _{t\in T}d_{jj'}^{u}\cdot O_{jj'l}^{ut}\\&\quad + \sum _{u\in U}\sum _{k\in K^{u}}\sum _{t\in T} \varGamma \cdot Z_{k}^{ut} \end{aligned} \end{aligned}$$

Equation 1 minimizes the total cost of the transportation planning, that is, the cost of the routing, the cost of the long-haul transportation and the cost for using LDTs.

subject to

2 $$\begin{aligned}&\sum _{i'\in V^{u}}\sum _{k \in K^{u}}\sum _{t \in \left[ E_{i},A_{i}\right] }X_{ii'k}^{ut} = 1, \; \forall u\in U, i\in V^{u} \end{aligned}$$

3 $$\begin{aligned}&\sum _{i\in V^{u}}X_{j_{0}^{u},ik}^{ut} = \sum _{i\in V^{u}}X_{i,j_{1}^{u},k}^{ut} \le 1, \; \forall u\in U, j\in P^{u}, k\in K^{u}, t\in T \end{aligned}$$

4 $$\begin{aligned}&\sum _{i'\in V^{u}} X_{ii'k}^{ut} - \sum _{i'\in V^{u}}X_{i'ik}^{ut} = 0, \;\forall u\in U, i\in N^{u}, k\in K^{u}, t\in T \end{aligned}$$

This block of constraints corresponds to the flow constraints in the routing part. Constraint (2) assures that a certain node (pickup or delivery point) in a region is assigned to one and only one route within the earliest and latest day of service of its corresponding request ($\left[ E_{i},A_{i}\right]$). Constraints (3) force every route to start and to end in the same depot of the corresponding region, while the flow conservation constraints are given in (4). The latter ensure that if a node is visited by vehicle *k*, this same vehicle also leaves the node.

5 $$\begin{aligned} Q_{i'k}^{ut}&= \sum _{i\in V^{u}}\sum _{t\in T}X_{ii'k}^{ut} \cdot \left( Q_{ik}^{ut} + q_{i}^{u}\right) , \; \forall u\in U, i'\in V^{u}, k\in K^{u} \end{aligned}$$

6 $$\begin{aligned}&\quad 0 \le Q_{ik}^{u} \le Q_\mathrm{SH}, \; \forall u\in U, i\in V^{u}, k\in K^{u} \end{aligned}$$

Constraints for the load of LDTs. Term (5) models the evolution of the load in a route, while (6) keeps the load below the limit.

7 $$\begin{aligned} S_{i'k}^{ut} = \sum _{i\in V^{u}}\sum _{t\in T}X_{ii'k}^{ut} \cdot \left( S_{ik}^{ut} + c{ii'}^{u}\right) , \; \forall u\in U, i'\in V^{u}, k\in K^{u} \end{aligned}$$

This group of equations model the times of service for each node. Term (7) ensures that the service times are consistent within a short-haul route.

8 $$\begin{aligned}&\sum _{i'\in V^{u}} X_{ii'k}^{ut} - \sum _{i'\in V^{u}}X_{i+|R^{u}|,i'k}^{ut} - \sum _{i'\in V^{u}}\sum _{k\in K^{u}}\sum _{t'\in T,t'>t} X_{i+|R^{u}|,i'k'}^{ut'} = 0, \;\nonumber \\&\quad \forall u\in U, i \in R^{u}, k \in K^{u}, t \in [E_{i},A_{i}] \end{aligned}$$

9 $$\begin{aligned}&S_{ik}^{ut} \le S_{i+|R^{u}|,k}^{ut}, \; \forall u\in U, i\in R^{u}, k\in K^{u}, t\in T \end{aligned}$$

These equations model the constraints for intra-region requests. Equation (8) assures that if the pickup node *i* of an intra-region request is visited by vehicle *k* in day *t*, then the delivery node $i+|R^{u}|$ is visited by the same vehicle on the same day or by any vehicle in later days. Equation (9) guarantees that if an intra-region request is served in a single route, the delivery point is scheduled later in the route. This would correspond to the typical precedence constraint in the classical PDP.

10 $$\begin{aligned} \sum _{i,i' \in V^{u}} X_{ii'k}^{ut} \le M\cdot Z_{k}^{ut}, \forall u\in U, k\in K^{u}, t \in T \end{aligned}$$

Constraints 10 refer to the usage of LDTs. If a route is performed by vehicle *k* in region *u* and day *t*, this vehicle is set as used and accounted for in the total cost.

11 $$\begin{aligned} \begin{aligned}&\sum _{i'\in V^{u}}\sum _{k\in K_{j}^{u}}X_{ii'k}^{ut^{*}} = \sum _{j'\in P^{{\bar{u}}}}\sum _{l\in L_{j}^{u}}\sum _{t^{*} \le t < A_{i}} Y_{ijj'l}^{ut}, \; \\&\quad \forall u\in U, i\in N^{u}, j \in P^{u}, t^{*} \in T \end{aligned} \end{aligned}$$

12 $$\begin{aligned} \begin{aligned}&\sum _{j\in P^{u}}\sum _{l\in L_{j}^{u}}Y_{ijj'l}^{ut^{*}} = \sum _{i'\in V^{{\bar{u}}}}\sum _{k\in K_{j'}^{{\bar{u}}}}\sum _{t^{*}<t \le A_{i}} X_{ii'j'k}^{{\bar{u}},t^{*}}, \;\\&\quad \forall u\in U, i\in N^{u}, j'\in P^{{\bar{u}}}, t^{*}\in T \end{aligned} \end{aligned}$$

The last two constraints connect variables *X* and *Y*. Constraints (11) ensure that a request departs on a long-haul trip from the same depot that services the pickup by an LDT. Equivalently, (12) make sure that the short-haul route delivering the goods of a request, departs from the same depot where the HDV transporting the request arrived.

13 $$\begin{aligned} \sum _{i\in N^{u}}Y_{ijj'l}^{ut} \le M\cdot O_{jj'l}^{ut} , \; \forall u\in U, j\in P^{u}, j'\in P^{{\bar{u}}}, t\in T \end{aligned}$$

Constraint (13) connects variables *Y* and *O*. If any request is transported in HDV *l* between depots *j* and $j'$ then this vehicle is used.

14 $$\begin{aligned} \sum _{i\in N^{u}}Y_{ijj'l}^{ut}\cdot D_{i} \le Q_\mathrm{LH}, \forall u\in U, j\in P^{u}, j'\in P^{{\bar{u}}}, l\in L_{j}^{u}, t\in T \end{aligned}$$

Last term (14) makes sure the load on an HDV does not exceed the specified limit.

15 $$\begin{aligned} \begin{aligned}&X_{ii'jr}^{ut},\quad Y_{ijj'r}^{ut},\quad O_{jj'l}^{ut} \in \{0, 1\}\\&Q_{ik}^{ut},\quad S_{ik}^{ut},\quad W_{i}^{u} \in Z \end{aligned} \end{aligned}$$

The model is implemented using a commercial solver, but only small instances were solved to optimality within a predefined time of 3 hours (see Sect. 5). Therefore, we propose an heuristic approach for tackling bigger instances that could be found in real life situations.

## Methods

For solving such a complex problem as the 2R-MDPDP, we make use of an ALNS algorithm. This is a widely used metaheuristic framework that has been adapted to solve several optimization problems. The choice of ALNS is motivated by its good performance for VRP and in particular for two-level decision problems. It was firstly proposed by Ropke and Pisinger (2006) for the PDP with time windows and lately extended in Pisinger and Ropke (2007) to several VRP classes. Results show very good performance and high consistency in all instances solved. Regarding two-level decision settings, this method has been successfully applied to different problems. Ghilas et al. (2016a) use an ALNS approach for the PDP with time windows and scheduled lines. Similarly, Hemmelmayr et al. (2012) successfully tackle a two-echelon VRP with city logistics constraints using an ALNS algorithm. Kovacs et al. (2012) apply ALNS to a service technician routing problem. Results in all cases outperform or stay close to best-known results.

The main characteristics of ALNS are inherited from the family of large neighborhood search (LNS) algorithms. These algorithms perform a search through the solution space by iteratively generating new solutions using a ruin and recreate scheme. In each iteration, part of the solution is destroyed and subsequently repaired. The new solution obtained is kept as new incumbent solution if certain acceptance criteria are met. The destroy and repair strategies are guided by different operators designed and adapted for the problem at hand. Normally, the choice of the operators to use in each iteration is performed randomly. The adaptive variant of these algorithms consists of introducing a learning component for a more intelligent selection of operators. A scoring scheme keeps track of the pairs of operators that historically generate better solutions. The destroy–repair combinations with higher scores have a higher probability of being used in subsequent iterations.

The general framework of the ALNS algorithm presented in this paper starts with an initial solution, which is set as incumbent solution *x* for beginning the search, with cost *f*(*x*). Then a pair of destroy and repair operators is selected and a new solution $x'$ with cost $f(x')$ is generated. If this solution is better than the best obtained solution $x^{*}$, $x'$ replaces $x^{*}$ and is the new incumbent solution for the next iteration. In case $f(x') \ge f(x^{*})$, $x'$ can still be kept as incumbent solution if one of the following criteria is met:

- *Acceptance criteria 1* New solution $x'$ is accepted as new incumbent solution with probability $e^{(f(x) - f(x'))/T}$. Parameter *T* controls this probability of acceptance along the search. It is initialized with value $T_{0} = 0.05\times f(x)\times \log (0.5)$ and updated by a decreasing factor of $T\_\mathrm{cooling}$ after each iteration.
- *Acceptance criteria 2* The new solution $x'$ is also accepted as new incumbent solution in case that $f(x') < f(x) + 0.5$. This helps to maintain a path toward a local optima when a new incumbent solution is accepted with acceptance criteria 1.

If none of these acceptance criteria are met, the new solution $x'$ is rejected and a new iteration starts keeping *x* as incumbent solution. Regarding the scoring system for each pair of destroy–repair operators, three different scores *z*1, *z*2, *z*3 are used, depending on the new solution performance. If *x* improves the best solution, the score of the used destroy–repair pair of operators is rewarded with quantity *z*1. In case that acceptance criteria 2 is met, the selected pair of operators is rewarded with *z*2. In case *x* is accepted due to acceptance criteria 1, *z*3 is added to the score of the pair destroy–repair that led to this new solution. For the case when *x* is rejected, no score reward is granted for that pair of operators. During the algorithm process, the selection probabilities for each destroy–repair pair are updated after a batch of iterations. This is done by using the accumulated scores of each pair during the last batch (*PairOp*.*Score*), the number of times the pair has been selected during this same batch (*PairOp*.*Count*) and the overall performance value of each operator pair, which is updated as $PairOp.Perf = 0.5\times PairOp.Perf + 0.5\times (PairOp.Score/PairOp.Count$). With these values at hand, the selection probabilities of each pair (*PairOp*.*Prob*) are updated proportionally to the performance value of the pairs as $PairOp.Prob = PairOp.Perf / \sum _\mathrm{pairs}PairOp.Perf$. The frequency of this update is controlled by parameter *frequencyUpdate*, which determines the number of ALNS iterations performed before a new update on the probabilities is done. Values *PairOp*.*Score* and *PairOp*.*Count* are set to 0 after each probability update. In Sect. 5, we present results for the tuning process of the most relevant parameters involved in the algorithm. Operators are conceptually based on existing ones from the works of Pisinger and Ropke (2007) and Hemmelmayr et al. (2012) and adapted to each of the subproblems solved by the integrated algorithm.

### ALNS operators

As highlighted in Table 1, the 2R-MDPDP is composed of three different decision problems. Therefore, when designing an ALNS algorithm for tackling it, a good strategy for handling the three problems simultaneously must be found. For this purpose, we develop three kinds of destroy and repair operators for generating a new solution:

1. Operators for the scheduling problem. These operators work on the assignment of inter-region requests to HDVs and the integration of its customers in the routing plan of the depots between which the request is transported.
2. Operators for the multi-depot PDP. The operators of this kind work exclusively on the intra-region requests. They focus on the assignment of requests to depots and the routing of their pickup and delivery customers. Each region can be treated separately.
3. Operators for the VRPMB $+$ PDP. These operators work at a depot level and focus solely on the improvement of the routing plan for the given depot in all days.

**Fig. 3:**
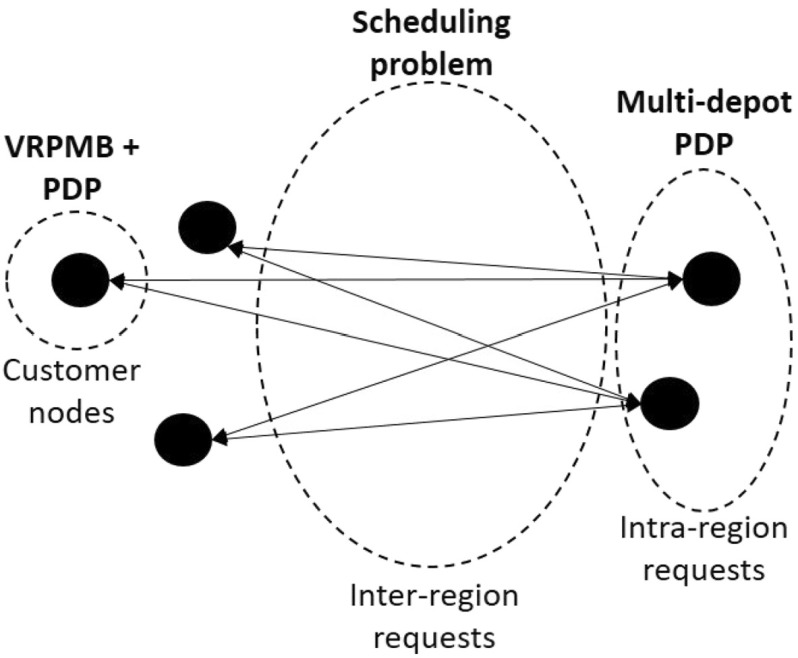
The three problems arising within the 2R-MDPDP. Each black node represents a depot

When selecting a destroy–repair pair in each iteration of an ALNS, a pair belonging to one of these three operator types is selected. In any case, a destroy operator from one kind can be coupled with a repair operator of another kind, since the elements these operators deal with, are of different nature. In each iteration, a destruction rate is randomly chosen between 10 and 35%. This rate is applied to the environment in which the operators work with. For example, a destroy operator from the scheduling problem type will remove between 10 and 35% of the total number of inter-region requests, while leaving the elements of the other problems unchanged. In the next sections, a detailed explanation for each type of operators is provided. Figure 3 gives a graphical description of the three different problems and shows to which parts of the solution they are related.

#### Operators for the scheduling problem

Making changes on the assignment of inter-region requests to HDVs also affect the routing decisions, since a removal/insertion of an inter-region request from the solution also implies a removal/insertion of its customer nodes from/in an LDT in their respective regions. Therefore, when these operators are applied they must guarantee: (i) routing feasibility conditions (see Sect. 1); (ii) pickup and delivery nodes of each inter-region requests are served from the depots between which the HDV is transporting the request; (iii) days of long-haul transportation must be consistent with the days that pickup and delivery nodes are serviced. The algorithm makes use of 5 destroy operators and 3 repair operators.

**Destroy operators**

- *Removal random inter* Inter-region requests are randomly removed.
- *Removal worst inter* This operator selects those inter-region requests that generate a higher cost on the solution. The cost of an inter-region request in the solution is calculated as the proportional cost of this request in the HDV and the cost of its customers in their respective short-haul routes, $C_{r} = C^\mathrm{LH}_{r} + C^\mathrm{SH}_{r,p} + C^\mathrm{SH}_{r,d}$, where indices *p* and *d* stand for pickup an delivery customer, respectively. The cost proportion of request *r* in its HDV is calculated as the share of *r* in the total quantity transported by the vehicle, $C^\mathrm{LH}_{r} = \mathrm{LHCost} \times (r_\mathrm{demand}/LH_\mathrm{quantityTransported})$. The cost of a customer node of *r* in its short-haul route is the difference in distance between the current route and the same route without this customer. In each removal step, a list of the requests with higher cost is generated and sorted. To randomize the search, one of the inter-region requests is selected following a probability rule from the top of the list.
- *Removal related inter* This removal strategy seeks to remove requests that show a high similarity. The idea is that removing requests considered similar would lead to a highest degree of flexibility and interchange in the later repair step. Similarity between requests *r* and $r'$ is measured by a relatedness score $R_{rr'}$ calculated as a combination of two factors: geographical distance and due date distance. The first one is the distance between the pickup and delivery customers of the requests, $D_{rr'} = d_{r_{p}r'_{p}} + d_{r_{d}r'_{d}}$. The due day distance is the difference in the due dates for servicing each request, $P_{rr'} = |E_{r} - E_{r'}| + |A_{r}-A_{r'}|$. Both distance measures are normalized by dividing the obtained raw value by the maximum possible distance, in terms of geographical locations and due dates. Finally, $R_{rr'} = D_{rr'} + P_{rr'}$. The operator starts by randomly selecting a request to remove from the route. In each step, the relatedness value is computed between the last removed request and each request in the solution. The request with highest score is then removed. The process continues until the desired number of removed requests is reached.
- *Removal HDV inter* In this operator, entire HDVs and their transported requests are removed from the solution. To avoid an excessive rate of destruction in the solution, the destruction rate is selected randomly between 10 and 20% of the total number of HDVs. Inside the operator, two removal strategies are defined. In the first one, a vehicle is randomly selected and removed. The second strategy removes vehicles incurring higher costs. The costs for a vehicle *l* are measured as the average costs of all its transported requests, $C_{l} = \sum _{r\in l} C^\mathrm{LH}_{r}/|l|$, where |*l*| is the number of requests transported by *l*. In each step, one of these strategies is selected with probability 50%.
- *Removal proximity inter* This operator prioritizes the removal of inter-region requests whose customers are close to other depots in the corresponding region. The goal is to enhance the reallocation of requests to HDVs traveling to depots where the customers of the request might find a good routing position.

**Repair operators**

- *Repair greedy insertion inter* In this operator, the reinsertion of removed inter-region requests follows a greedy strategy. For each request in the reinsertion pool, the insertion position with the least cost is calculated. The cost of insertion of a request to HDV *l* is calculated using the same equation as in the *removal worst* operators. That is, the shared cost of the request if transported by vehicle *l* plus the cost derived from adding the customers to an existing route or from creating a new route. When a new route is created, a fixed cost per vehicle is added to the distance cost. To decrease the deterministic outcome of the method, we create a list with all requests best insertion positions and randomly select one of the top. The process is repeated until all requests have been assigned to an HDV.
- *Repair regret inter* The k-regret insertion operators aim at selecting those requests with a bigger difference between the cost of the best insertion position and that of the k-best one. The idea behind it is that not inserting a request with a big cost difference might lead to bad insertion afterward. The costs are calculated in the same way as in the *repair greedy insertion* operator. Also a list of all requests along with their regret value is created and a request is randomly chosen from the top of the list. Our ALNS algorithm makes use of 2-regret and 3-regret operators.

#### Operators for the multi-depot PDP

In the multi-depot PDP problem, we must solve the assignment of the intra-region requests to a depot as well as the service of this request in short-haul routes from that depot. As described in Sect. 3, we consider the possibility of servicing the pickup and delivery customers of an intra-region request on different days. Therefore, precedence constraints must hold only if the request is served on one day, in which case it is served by the same LDV. To deal with this problem 3 removal operators along with 3 repair operators are used. In each iteration, we remove full intra-region requests from the solution, as in the previously described operators for the scheduling problem.

**Destroy operators**

- *Removal random intra* In each step, a randomly chosen intra-region request is removed, until we reach the desired quantity of requests to remove.
- *Removal worst intra* This operator works in the same way as *removal random inter*. In this case, the cost of an intra-region request in the solution is calculated as the difference in distance cost in the routing with and without the request on it. Since the intra-region request has both customers in the routing plan of the same depot, cost $C_{r}$ is the cost that both nodes are generating in their corresponding routes, $C_{r} = C_{r,p} + C_{r,d}$.
- *Removal related intra* Again we use the same strategy as in the scheduling operator counterpart, *removal related inter*. The way of measuring the *relatedness* score between two requests is also identical, only that now we measure the scores of nodes situated in the same region.

**Repair operators**

- *Repair greedy insertion intra* This operator works similar to *repair greedy insertion inter*, but the costs are calculated as in *removal worst intra*. When inserting an intra-region request, all insertion positions in the routing plan that satisfy the due dates of the request, are evaluated. Afterward, the best combination of positions for the pickup and delivery nodes are selected, such that it satisfies the precedence constraints, if necessary.
- *Repair regret intra* Again we use a similar strategy to *repair regret inter.* Insertion costs and feasible positions are calculated as described in *repair greedy insertion intra.* 2-regret and 3-regret operators are used.

#### Operators for the VRPMB $+$ PDP

The operators designed for this problem work purely on the routing level at a single depot. That is, when a destroy–repair pair of this kind is selected, we apply it to every depot with the goal to optimize the routing plan over all periods. The starting solution in each depot is a set of routes for each day, which corresponds to the depot routing plan in the incumbent solution. The destroy operator then removes parts of the routed customers in the solution, creating a pool of non-inserted customers. These customers are then reinserted in the routing plan by means of the repair operator. This problem has two kinds of customers, according to the type of request they belong to:

1. The customers that belong to an inter-region request form the VRPMB. These customers are not paired with any other customer in the depot. Therefore, they do not need to satisfy precedence constraints, but they need to be coherent with the days in which their inter-region transport occurs. Delivery customers of this type impose an initial load on the vehicles leaving the depot. Their possible service days are determined by the day after which the HDV transporting these goods arrives, and the latest service day of the request. Pickup customers from inter-region requests are loaded during the route and dropped at the depot when the vehicle arrives. Their feasible days of service are between the earliest day of service of the request and the departure day of the HDV transporting the goods. These two days can be the same, which means that the only way for servicing this request is by picking up the goods during the day and sending them in the HDV in the evening.
2. The customers belonging to an intra-region request form a PDP problem. These customers are paired pickup and delivery nodes and must satisfy precedence constraints when served on the same day. If they are not served by the same route, their nature within the routing is similar to those belonging to inter-region requests described above.

In this problem, we only work on the customers, which means that no full inter-region or intra-region requests are removed. The pickup customer from an intra-region request can be removed while keeping the delivery customer in the routing plan. For tackling these two mixed problems, we devise 4 operators for solution destroying purposes and 3 for repair.

**Destroy operators**

- *Removal random sort-haul* This operator removes customers at random.
- *Removal worst short-haul* Similar to the previously described operators. In this case, the cost that a customer generates in a solution is calculated as the difference in distance cost in the route where it belongs, when comparing with the same route without the customer. If a customer is served in position *i* of a route, then $C_{i} = d(i-1,i) + d(i,i+1) - d(i-1,i+1)$, where *d*(*i*, *j*) is the distance between customers in positions *i* and *j* in the route. The customers with higher cost are removed from the solution following a randomized strategy as in the previous strategies.
- *Removal related short-haul* Same concept as *removal related inter*, but here the *relatedness* score between customers *s* and $s'$ is calculated as the sum of geographical distance $d_{ss'}$, customer due dates distance, and difference in visit time. The due date distance is calculated by the feasible days to serve a customer, in the same way as in *removal related inter*. As previously mentioned, feasible service days for customers from inter-region requests are determined by the due dates of the request and the day when the long-haul is transported. In case of customers from intra-region requests, the feasible service days are determined by the request’s due date and the day of service of the other customer of the request. That is, a delivery customer cannot be served on a day before the pickup occurs, and vice versa. The visit time distance is measured as $|b_{s} - b_{s'}|$, where $b_{s}$ is the visit time of customer *s*. The customer to remove in each round is selected identically as in the other removal related algorithms.
- *Removal route short-haul* This operator removes entire routes from the depot routing plan. As in *removal long-haul vehicle inter*, the removal rate is adjusted to 10–20% to avoid destroying too much. Also two different processes for removal can be used. One process removes a route randomly. The second process removes those routes with higher cost. The cost of route *v* is calculated as the average distance cost per customer in the route, $C_{v} = \sum _{i\in v} d(i,i+1)/|v|$, where |*v*| is the number of customers in route *v*. In each removal round, each process is selected with a probability of 50%.

**Repair operators**

- *Repair greedy insertion short-haul* The customers in the customer pool are reinserted in their cheapest feasible positions. The insertion cost is calculated in the same way as in *removal worst short-haul*. A fixed cost per vehicle is added to the distance cost when a new route must be created. A randomized selection of the best possible insertion positions is done in each round.
- *Repair regret short-haul* This operator works exactly in the same manner as the previously described regret operators. The cost structures are the same as in *removal worst short-haul*. We use 2-regret and 3-regret as insertion regret operators.

### Local search

Some local search operators are integrated within the ALNS procedures, with the objective of reaching solutions corresponding to local minima in the solution space. The search explores close neighboring solutions by performing small changes to the incumbent solution. The division of the 2R-MDPDP into 3 subproblems is also used for local search, where specific operators for the problem at hand are implemented. After each ALNS iteration, the search focuses only on the elements corresponding to the subproblem to which ALNS was applied. That is, inter-region requests for the scheduling problem, intra-region requests for the multi-depot PDP, and single customers for the VRPMB $+$ PDP. Nevertheless, the local search strategies used are the same for the 3 operators. These strategies are *Swap* and *Relocate*.

- *Swap* The swap strategy tries to interchange two elements from their current positions. In each iteration, a first element is randomly selected and all possible interchanges with other elements are evaluated until a feasible swap improving the objective value is found.
- *Relocate* This operator searches for relocations of randomly chosen elements to other places in the solution, providing that the change leaves the solution feasible and the move leads to an improvement in the objective function.

In order to limit the computational effort spent on local search moves, we stop the search after 250 iterations with no improving move.

Apart from the aforementioned local search procedure, we implement an additional local search to decrease the number of used short-haul vehicles and optimize their utilization by combining trips into the same vehicle. The combination of two trips is only possible if there is a starting time for both of them ensuring that no time window is violated, and depot opening times are respected. In this sense, we obtain a final solution for the 2R-MDPDP where all 3 subproblems are solved with the addition of multi-trip features in the LDT.

### Obtaining an initial solution

A feasible initial solution is needed as starting point for the ALNS. The given input data is the set of requests, along with the information of the depots in each region. The algorithm solves each subproblem in a greedy manner and in the following order:

1. *Inter-region request assignment (scheduling problem)* This step assigns all inter-region requests to an HDV in a greedy way. The cost of assigning a request to a lane is approximated by the sum of distances: (i) the two euclidean distances from the customer in each region to the corresponding depot of that lane in each region, and (ii) the distance between the two depots of the lane. Once we have all requests assigned to a long-haul lane and to the routing problem, we apply the two local search moves previously explained to optimize the assignment of inter-region requests to long-haul trips.
2. *Generate a feasible set of short-haul routes (VRPMB)* After the assignment is finished, we solve the routing with the customers from the inter-region requests. A sequential insertion algorithm based on Solomon I1’s algorithm (Solomon 1987) is used.
3. *Routing intra-region requests (multi-depot PDP)* In this case, a request can be inserted to any depot in the region where the request belongs to. A similar insertion algorithm as for the previous subproblem is used for routing these customers. Precedence constraints must be taken into account to ensure feasibility.

## Computational experiments

In the first part of our computational study, we assess the components of our ALNS based on state-of-the-art results for the considered subproblem (see Sect. 4).

### Algorithm performance for considered subproblems

Since no benchmark results for the 2R-MDPDP itself exist in literature, it is not possible to make a proper evaluation of the computational results obtained and presented in Sect. 5.5. However, we can assess the performance level of the proposed algorithm by applying it to each subproblem. Since we design ALNS and local search operators that work only on a subproblem level, we can also build separate algorithms to solve them individually. In this sense, we evaluate the performance of the algorithm on the subproblems of *inter-request scheduling problem* and *VRPMB*. We do that by solving two well-known problems in literature: VRPTW and LRP.

#### Results for the VRPTW

The classical VRPTW consists of finding a set of routes departing from a single depot, serving all customers within their time windows, and allowing for waiting time at the customers. All these customers are delivery nodes and are independent from each other, such that no precedence constraints are needed. The ALNS and local search operators devised for the *VRPMB* $+$ *PDP* subproblem can be used for the VRPTW. However, the difference lies in the fact that the elements of the algorithm related to customers belonging to an intra-region request (*PDP*) remain unused during the search, and the complexity of managing feasibility in the vehicle capacity constraints is lower due to all customers being delivery points. Computational tests are performed on Solomon VRPTW instances Solomon (1987) and compared to state-of-the-art benchmark results. Table 2 depicts the average results over 3 runs of the *VRPMB* $+$ *PDP* operators. Results show a good performance of the algorithm in terms of number of vehicles, matching the same values as state-of-the-art ones. Regarding routing costs, our algorithm is able to get all optimal solutions on instances C1 and C2, while staying between 1 and 2% away from best-known values in R1, R2, RC1, and RC2. We observe an average deviation from optimum of 1.04% on all instances. Taking into account that the operators are not specifically tuned for the VRPTW, we can argue that the tested operators work sufficiently well to guarantee a good performance on the routing level.

**Table 2:** Results for the *VRPMB* $+$ *PDP* subproblem operators on the VRPTW instances

	Benchmark		ALNS for VRPMB $+$ PDP	
	nVeh	z	nVeh	Gap (%)
C1	10	828.38	10	0
C2	3	589.85	3	0
R1	11.91	1210.33	11.91	1.54
R2	2.72	951.03	2.72	1.79
RC1	11.5	1384.16	11.5	1.33
RC2	3.25	1119.24	3.25	1.58

#### Results for the LRP

Computational experiments on LRP aim at assessing operators for the *inter-region requests scheduling* and the *VRPMB* subproblems. LRP benchmark instances can be transformed into 2R-MDPDP instances through the following steps:

- The first region contains only the hub as a depot, while the second one contains all possible depot locations for the LRP instance.
- All LRP requests are transformed into inter-region requests, all of them with the pickup node located in the LRP hub and the delivery node being the customer location in the LRP instance.
- Only one long-haul vehicle per lane is allowed. The capacity of the long-haul vehicle is the capacity of the potential depot in the region, where it arrives. The cost of the long-haul trip is the opening cost of this depot.

The generated 2R-MDPDP has no intra-region requests. Therefore, PDP elements are not used during the solution procedure, as in the previous case for the VRPTW. The scheduling problem works on the allocation of inter-region requests to long-haul vehicles, which translates into the assignment of the request delivery point to a potential depot. If any request is assigned to a depot, the opening cost of the depot is added to the objective value through the HDV cost.

For testing our algorithm, we solve the instances proposed by Barreto (2004) and Prins et al. (2006) with capacities on the depots, and by Tuzun and Burke (1999) without depot capacities. We compare our results to state-of-the-art solutions, but also to the work of Hemmelmayr et al. (2012). In this paper, the authors present an ALNS algorithm for the two-echelon routing problem and the LRP. To the best of our knowledge, this is the only attempt to solve an LRP with an ALNS algorithm, so it is an important benchmark for the evaluation of our algorithm. Table 3 shows the average results obtained for the LRP tests, with 3 runs per instance. More detailed results can be found in the “Appendix”. It can be seen that, despite not being specifically designed for this kind of problems, our proposed algorithm stays very close to best-known solutions and to the results taken from the work of Hemmelmayr et al. (2012). Hence, we can conclude that the tested operators have a sufficiently good performance.

**Table 3:** Gap to state-of-the-art ALNS for the LRP

	H (%)	Scheduling $+$ VRPMB (%)
Barreto	0.21	0.42
Prins	0.79	0.42
Tuzun	0.57	0.74

H refers to the work of Hemmelmayr et al. (2012)

### Experimental data

Since the 2R-MDPDP has not been previously addressed in the literature, no available instances for this problem exist. Therefore, new instances for solving the problem were generated. The data used in this paper is publicly available^1^. Each instance has 5 main elements: (i) number of inter-region requests, (ii) number of intra-region requests, (iii) number of depots, (iv) number of days and (v) vehicle capacities. Instances have been randomly generated with some predefined values for these main characteristics. We generate two sets of instances, one with time windows on the customer nodes (set T) and another one without time windows (set N). Table 4 provides a summary of all instance characteristics. Figure 4 shows an example of an instance with 25 inter-region requests, 25 intra-region requests and 2 depots in each region.

**Table 4:** Instance characteristics summary

Instance	#Inter-region requests	#Intra-region requests	#Depots	#Days	Vehicle capacities
T01	50	50	2/2	4	900/2000
T02	25	50	3/3	3	1800/4000
T03	75	25	2/2	3	1800/4000
T04	25	25	2/3	4	1800/4000
T05	25	50	2/2	3	1800/4000
T06	50	75	3/3	2	3600/8000
T07	25	50	3/3	2	3600/8000
T08	50	25	2/2	3	3600/8000
T09	50	25	2/2	2	3600/8000
T10	75	50	3/3	3	1800/4000
T11	75	25	2/2	3	900/2000
T12	25	75	2/2	3	1800/4000
N01	75	50	3/3	2	1800/4000
N02	75	75	3/3	3	900/2000
N03	75	75	3/3	4	900/2000
N04	75	25	3/2	4	900/2000
N05	50	75	2/2	4	3600/8000
N06	75	50	3/2	2	1800/4000
N07	50	25	3/2	3	900/2000
N08	25	25	3/3	3	3600/8000
N09	75	50	2/3	2	900/2000
N10	50	50	2/3	4	3600/8000
N11	50	25	2/2	3	3600/8000
N12	50	50	2/2	4	900/2000

#depots refers to region1/region2. Vehicle capacities indicate short-haul/long-haul capacities

**Fig. 4:**
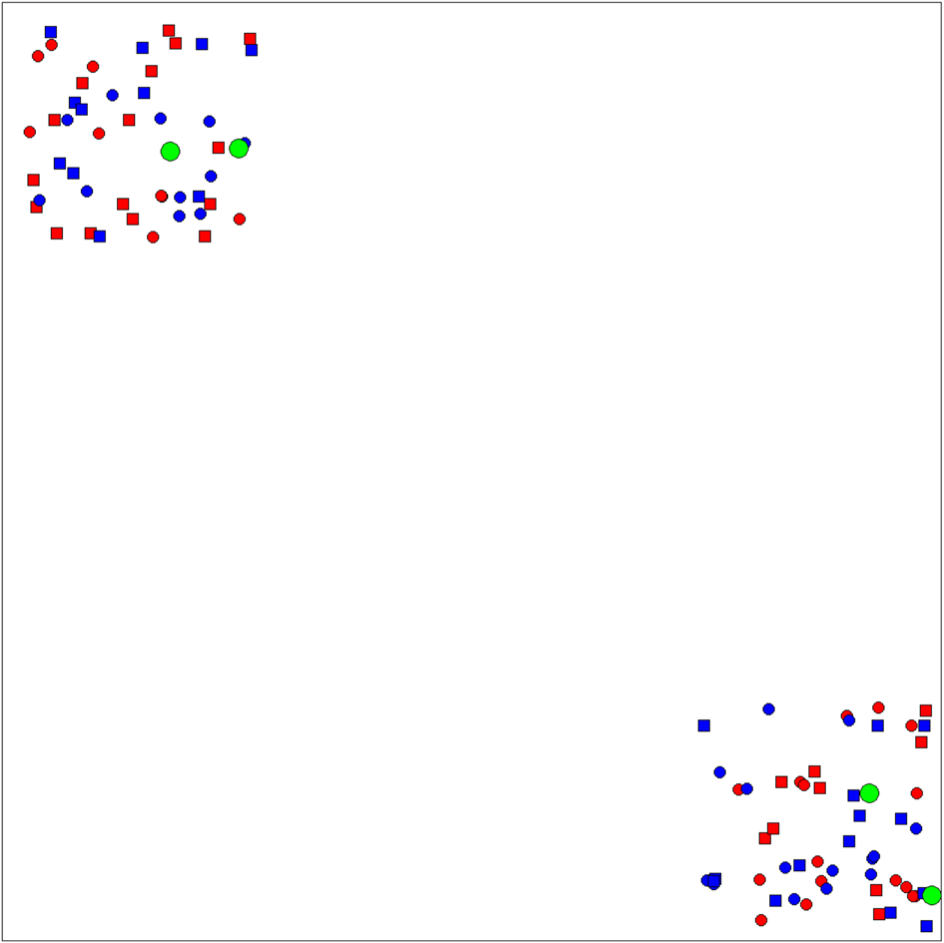
Representation of a data instance with 25 requests of each kind and two depots in each region. Blue and red nodes represent inter-region and intra-region requests, respectively. Squares and circles represent pickup and delivery customers (color figure online)

### Parameter tuning

In order maximize the performance of the proposed ALNS algorithm, we tune the most relevant parameters of the algorithm. For this purpose, we use the iterated racing method proposed in López-Ibáñez et al. (2016) through the existing *irace* R library. This methodology receives as inputs the set of parameters from the algorithm to be tuned, together with their possible set of values and a set of test instances *I*. Normally, the larger the sets of parameters and test instances provided, the more accurate the tuning can be. However, large sets also imply the need for very large computational effort. They can end up with incomplete performances of the *irace* algorithm if the computational budget is limited. Therefore, we made a selection of the most relevant parameters and generated a minimal set of instances that we could consider highly representative. For the instance set, we generate 16 instances, 8 with time windows and 8 without time windows. In each subset, we find instances from varying sizes (from 20 to 200 requests), varying number of depots (2,3) and varying capacities as described in Sect. 5.2. The number of periods is set to 3 in all instances. Regarding the parameters, we considered 5 main parameters. Table 5 shows the selected parameters together with their possible values.

After the racing process, the *irace* algorithm returns three possible parameter configurations for which no statistical evidence was found regarding their performance: {8, 6, 2, 0.992}, {8, 5, 2, 0.997} and {7, 5, 1, 0.995}. The computational tests described in the next sections were performed using configuration *z*1 = 8, *z*2 = 5, *z*3 = 2, *T* = 0.997. Apart from those, we set the parameters $T\_\mathrm{cooling}$ = 0.9999 and *frequencyUpdate* = 100.

**Table 5:** Parameters to be tuned and possible values

Parameter	Range value
*z*1	{10, 9, 8, 7}
*z*2	{6,5,4}
*z*3	{3,2,1}
*T*	[0.95, 0.9999]

### MIP tests

Some tests for the MIP formulation (Sect. 3) were run for tiny instances. The goal of these runs were to (i) assess the size of the instances we were able to solve within 3 hours of computational time, and to (ii) compare the performance of the ALNS algorithms against the optimal solutions. For these experiments, we generated 2 sets of 10 small instances (with and without time windows) with 5, 7 and 9 inter-region and intra-region requests, 2 depots per region, and 3 days. The largest instances that we were able to solve to optimality have 9 inter-region and 7 intra-region requests. Experiments were performed using the commercial solver ILOG CPLEX 12.6.3, on a 3.6 GHz computer with 8 GB RAM. Additionally, we solve these instances with the proposed ALNS approaches to test the performance of the designed algorithms on small instances. In Table 6, we report the solutions found for the MIP formulation together with the optimality gap and the corresponding lower bound result. We compare this lower bound value with the average results obtained for 3 runs of 10 seconds each by our ALNS algorithm. We see that the ALNS approach finds optimal solutions in all instances, where CPLEX provides a solution. In all others, we stay close to the lower bound. This can be seen as an indicator that probably the optimal solution is also reached. As a remark, it seems that the number of intra-region requests plays a bigger role in the problem complexity than the number of inter-region requests. As can be seen, cplex is able to find the optimal solution when the number of intra-region requests is low. For the case of instances with time windows (TS), cplex solves any instance with less than 9 intra-region requests, with generally high lower bound gaps on the non-solved instances. Regarding the instances with no time windows (NS) an optimal solution is only reached when the number of intra-region requests is 5, but the lower bound gap for the other instances is lower than in the TS case. This could be explained by the smaller solution space that the TS instances have due to time windows restrictions.

**Table 6:** Results found by CPLEX (column *MIPSol*) and ALNS

	#Req	MIPSol	%OptGap	LB	ALNS	%Gap
TS00	9/9	5341.04	12.8%	4657.39	4752.56	2.0%
TS01	7/9	5952.54	13.1%	5175.14	5336.05	3.1%
TS02	9/7	4699.87	0.0%	4699.87	4699.87	0.0%
TS03	9/7	5915.72	0.0%	5915.72	5915.72	0.0%
TS04	7/5	3489.58	0.0%	3489.58	3489.58	0.0%
TS05	9/9	3839.40	18.8%	3117.59	3196.39	2.5%
TS06	7/9	3335.60	16.2%	2795.23	2877.05	2.9%
TS07	7/9	5363.90	23.5%	4103.38	4163.10	1.5%
TS08	5/9	2298.82	4.5%	2195.37	2218.41	1.0%
TS09	5/7	3018.70	0.0%	3018.70	3018.70	0.0%
NS00	7/7	4127.63	0.0%	4127.63	4127.63	0.0%
NS01	9/7	4671.66	8.1%	4293.26	4296.64	0.1%
NS02	9/9	4637.60	8.6%	4238.77	4326.73	2.1%
NS03	9/7	5090.58	8.2%	4673.15	4741.16	1.5%
NS04	5/5	2922.36	0.0%	2922.36	2922.36	0.0%
NS05	9/9	4676.25	5.1%	4439.63	4484.16	1.0%
NS06	5/7	4694.72	4.1%	4504.11	4549.69	1.0%
NS07	5/7	3065.51	2.2%	2998.07	3065.51	2.2%
NS08	5/5	3770.51	0.0%	3770.51	3770.51	0.0%
NS09	5/7	3881.00	5.9%	3652.02	3664.38	0.3%

For CPLEX we report optimality gap (*%OptGap*) and lower bound (*LB*). Each run of the ALNS algorithm is terminated after 10 s

*#Req* show the number of inter-region and intra-region requests respectively

### Experimental results

**Fig. 5:**
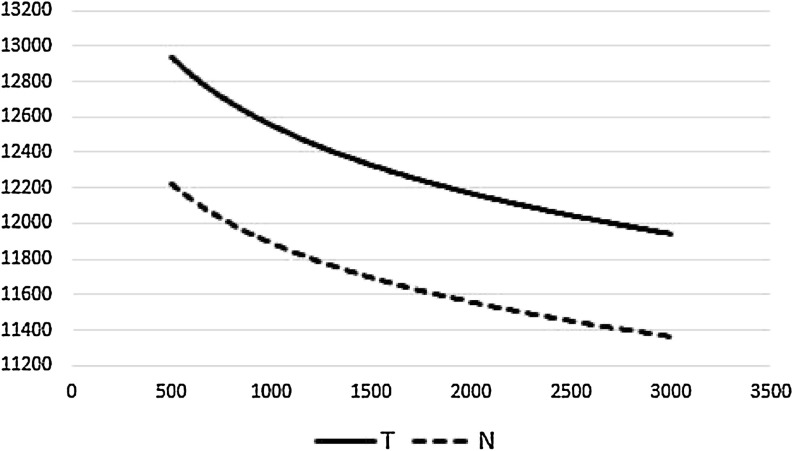
Evolution of solution quality with the value of *noImprovement* iterations

In this section, we discuss the computational results obtained for the instances described in Table 4. For obtaining computational results, the parameter settings described in Sect. 5.3 have been used. The stopping criteria for each run of an instance is controlled by the parameter *noImprovement*, which is the number of consecutive iterations without solution improvement. Every time a new incumbent solution is found, *noImprovement* is set to 0. We perform experiments for several values of *noImrpovement*: 500, 750, 1100, 1500, 2200 and 3000. By obtaining solutions for different values, we want to test the convergence behavior of the method and evaluate the impact of higher values for *noImrpovement* on solution quality. This kind of analysis has an interesting managerial insight, since it gives an idea of the additional computational effort necessary to reach a certain solution quality level. Table 8 shows the average results over 3 runs of each instance for *noImprovement* set to 500, 1500, and 3000. Results show a decreasing tendency in the total cost when experiments with a higher number of iterations are performed. However, it can be seen that this tendency is not linear. Figure 5 shows the graphical representation of the average costs as a function of parameter *noImprovement* for sets *T* and *N*. This highlights the importance of knowing the price (in terms of computational effort) of improving solution quality. As an example, Table 7 shows the evolution of gains in solution quality for the values of *noImprovement* (Table 8). The values show how much the total cost is reduced for each second of additional computational effort. A significant difference can be evidenced between both ranges. A practitioner trying to solve the 2R-MDPDP may consider worth the effort to run the algorithm for *noImprovement* set to 1500 but not further.

**Table 7:** Price of solution quality for *noImprovement* set to 500, 1500 and 3000

	500–1500	1500–3000
*T*	14.86	2.41
*N*	3.81	0.35

**Table 8:** Detailed results obtained for *T* and *N* instances: total cost (*totCost*), total distance (*totDist*), number of long-haul vehicles (*lhVeh*), number of short-haul vehicles (*shVeh*), computational time in seconds (*cpu*)

	500 iter					1500 iter					3000 iter				
	totCost	totDist	lhVeh	shVeh	cpu(s)	totCost	totDist	lhVeh	shVeh	cpu	totCost	totDist	lhVeh	shVeh	cpu
T01	20510.79	6844.12	9.33	27.33	27	19779.37	6446.03	8.33	26.67	79	18072.18	6238.85	7.00	23.67	455
T02	9721.03	3554.36	3	12.33	30	8957.17	3290.51	3.00	11.33	95	8938.48	3271.81	3.00	11.33	185
T03	12051.27	4717.94	4.67	14.67	16	11344.59	4511.26	5.00	13.67	56	11388.09	4388.09	4.67	14.00	113
T04	9820.73	3654.07	5	12.33	5	9095.25	3595.25	5.33	11.00	19	8625.37	3458.71	4.67	10.33	61
T05	12425.67	4092.34	3.67	16.67	21	11893.10	4059.77	3.33	15.67	51	11726.58	3893.25	3.00	15.67	136
T06	12881.36	4548.02	2.33	16.67	116	12684.78	4518.11	2.33	16.33	334	12680.61	4513.94	2.67	16.33	802
T07	9454.35	3621.01	3.33	11.67	26	8784.60	3117.93	2.00	11.33	78	8840.46	3173.80	2.00	11.33	213
T08	9262.36	3762.36	4.33	11	14	9018.51	3518.51	4.00	11.00	37	9018.09	3684.76	4.33	10.67	49
T09	7312.97	2979.64	2	8.67	11	7000.82	2834.15	2.00	8.33	34	6833.32	2833.32	2.00	8.00	69
T10	17784.05	6284.05	7.67	23	35	15912.66	5745.99	5.67	20.33	204	15492.85	5659.51	5.33	19.67	361
T11	19826.98	6160.31	8.33	27.33	21	19484.60	5984.60	8.67	27.00	57	19312.62	5645.95	8.00	27.33	105
T12	14508.28	4841.62	4.33	19.33	47	13258.25	4758.25	4.00	17.00	276	13264.96	4431.62	4.00	17.67	469
N01	11742.47	3909.14	4.67	15.67	223	11797.69	3797.69	4.00	16.00	605	11609.95	3776.61	4.33	15.67	1131
N02	19493.80	6827.14	9.67	25.33	203	19180.04	6680.04	9.67	25.00	1344	18917.06	6583.73	10.00	24.67	3813
N03	20113.55	7280.22	12.67	25.67	189	18793.12	6959.79	12.33	23.67	1368	18116.34	6616.34	9.33	23.00	2767
N04	18233.43	6233.43	10	24	19	16880.34	5713.67	8.67	22.33	106	17119.52	5786.19	9.33	22.67	184
N05	7386.80	3386.80	5	8	557	7352.54	3352.54	5.00	8.00	1358	7327.85	3327.85	5.00	8.00	2990
N06	10558.65	3558.65	4.33	14	180	10354.32	3354.32	4.00	14.00	446	10276.74	3276.74	4.00	14.00	1576
N07	13647.31	4813.98	7.67	17.67	13	12598.68	4432.02	7.33	16.33	66	12242.38	4075.72	6.33	16.33	191
N08	5218.46	2218.46	4	6	21	5169.43	2169.43	4.00	6.00	43	5174.43	2174.43	4.00	6.00	120
N09	20560.64	6393.97	8	28.33	134	20259.34	6259.34	8.33	28.00	407	20329.00	6329.00	8.67	28.00	1411
N10	7835.87	3669.20	6	8.33	112	7543.43	3543.43	6.00	8.00	461	7524.39	3524.39	6.00	8.00	1568
N11	5415.58	2415.58	4	6	51	5424.67	2424.67	4.00	6.00	133	5393.41	2393.41	4.00	6.00	501
N12	15057.63	5890.97	10.67	18.333	62	14258.97	5092.30	8.00	18.33	262	13974.41	5141.08	8.33	17.67	1523
Avg *T*	12963.32	4588.32	4.83	16.75	30.86	12267.81	4365.03	4.47	15.81	110.19	12016.13	4266.13	4.22	15.50	251.72
Avg *N*	12938.68	4716.46	7.22	16.44	146.99	12467.71	4481.60	6.78	15.97	550.09	12333.79	4417.12	6.61	15.83	1481.33

### Integrated versus sequential ALNS

Finally, we asses the benefits of the proposed ALNS to more classical approaches. As previously mentioned, we propose 3 kinds of operators each working on one of the identified subproblems of the 2R-MDPDP. We integrate all operators within the same framework such that all of them are applied “simultaneously”. However, a sequential approach can also be taken for constructing an ALNS algorithm with these different operator types. In transportation literature, there exist several problems with more than one decision level, where sequential algorithms are used to solve the problem. These algorithms focus on one of the decisions first and then optimize the other decision with the result obtained for the first one. The work of Côté et al. (2017) shows the benefits of designing an integrated algorithm that exploits the synergies between all decisions over a sequential one. In this sense, we design two sequential approaches to test against the proposed integrated approach. As previously described, the 2R-MDPDP can be split in three main problems that could be solved using different subproblem-related ALNS operators. Therefore, we can also design a sequential ALNS approach that fixes one of the decisions at a time. The first sequential algorithm starts with the higher level decision (scheduling of inter-region requests), then shifts the focus to the intra-region requests and finally ends with the lowest level (routing of customer nodes at a depot level). We refer to this algorithm as *Sequential ALNS High-Low*. The second approach is denoted as *Sequential ALNS Low-High* and does exactly the contrary to the previous one. For running the computational tests for each instance with the sequential algorithms, Termination criteria for each run on every instance with the sequential algorithms is the runtime of the same instance with the integrated ALNS (*noImprovement* $=\,1500$), that can be found in Table 8. This time is divided by 3 and each step of the sequential ALNS is implemented for that amount of time. Table 9 shows the average results over 3 runs on each instance of the sequential approach for both sets of instances, together with the gap to the integrated approach. We observe that there is a significant quality gap, especially for the instances with time windows, being the Sequential ALNS Low-High the worst of them on average.

**Table 9:** Comparison of integrated ALNS approach with sequential approaches

	Sequential ALNS high-low		Sequential ALNS low-high	
	totCost	%Gap	totCost	%Gap
T01	20701.49	4.66%	20625.99	4.28%
T02	9299.62	3.82%	9328.92	4.15%
T03	11755.35	3.62%	11744.46	3.52%
T04	9408.19	3.44%	9515.81	4.62%
T05	12386.38	4.15%	12188.26	2.48%
T06	13368.35	5.39%	13375.55	5.45%
T07	9055.37	3.08%	9256.22	5.37%
T08	9512.76	5.48%	9437.18	4.64%
T09	7218.12	3.10%	7237.97	3.39%
T10	16489.77	3.63%	16633.39	4.53%
T11	19927.84	2.27%	20358.03	4.48%
T12	13931.08	5.07%	13656.46	3.00%
Avg		3.96%		4.16%
N01	12059.05	2.22%	11961.04	1.38%
N02	19490.49	1.62%	19641.89	2.41%
N03	19224.32	2.29%	19229.47	2.32%
N04	17142.68	1.55%	17393.42	3.04%
N05	7483.01	1.77%	7520.79	2.29%
N06	10545.31	1.84%	10540.53	1.80%
N07	12883.76	2.26%	12855.45	2.04%
N08	5273.21	2.01%	5288.67	2.31%
N09	20557.74	1.47%	20837.07	2.85%
N10	7715.70	2.28%	7684.95	1.88%
N11	5525.45	1.86%	5532.99	2.00%
N12	14545.67	2.01%	14662.88	2.83%
Avg		1.93%		2.26%

The reported gap refers to the difference with respect with the result obtained with the integrated ALNS approach

## Conclusions

This paper presented a new class of problems in the context of pickup and delivery for large distribution networks, the 2R-MDPDP. The problem proposed in this work is a two-region variant with multiple depots in each region and a multiple day delivery scheme. It is composed of several interrelated decisions. A MIP formulation incorporating the main elements from multi-region problems, with the addition of multiple depot and multiple period settings, has been introduced.

We proposed a decomposition of the problem into three subproblems and used this decomposition to implement an ALNS algorithm framework integrating three kinds of destruction and repair operators. Each of them is dedicated to one of the subproblems. The dominance of the proposed method is shown by comparing it to a more classical ALNS approach, where the subproblems are treated sequentially.

Through a series of computational experiments, we proved the efficiency of the ALNS operators by comparing them to state-of-the-art results in their related subproblems. We also showed that the proposed framework is able to match exact solutions for small test sets. Benchmark instances and results for the proposed problem were provided. A study on the convergence of the algorithm through different values of stopping criteria provided an evaluation of the algorithm in terms of computational effort and solution quality.

This study presented one of the many possible variants for multi-region vehicle routing problems. Thus, a variety of related problems remains to be explored. For example incorporating concepts from other well-known problems like depot opening decisions, price collection problems or heterogeneous vehicles might be realistic extensions worth exploring. The proposed ALNS framework can be used to solve other multi-level decision problems in complex distribution networks.

## Appendix

See Tables 10, 11 and 12.

**Table 10:** Results for LRP on Barreto instances

Instance	BKS	H (%)	ALNS (%)
Christofides69-50$\times$5	565.60	0.00	0.00
Christofides69-75$\times$10	848.85	0.71	0.87
Christofides69-100$\times$10	833.40	0.24	0.36
Daskin95-88$\times$8	355.78	0.00	0.00
Daskin95-150$\times$10	43,919.90	1.31	2.16
Gaskell67-21$\times$5	424.90	0.00	0.38
Gaskell67-22$\times$5	585.11	0.00	0.00
Gaskell67-29$\times$5	512.10	0.00	0.00
Gaskell67-32$\times$5	562.22	0.00	0.00
Gaskell67-32$\times$5	504.33	0.00	0.00
Gaskell67-36$\times$5	460.37	0.00	0.32
Min92-27$\times$5	3062.00	0.00	0.00
Min92-134$\times$8	5709.00	0.41	1.35
	Avg	0.21	0.42

H refers to the work of Hemmelmayr et al. (2012)

**Table 11:** Results for LRP on Prins instances

	BKS	H (%)	ALNS (%)
20-5-1	54,793	0.00	0.00
20-5-1b	39,104	0.00	0.00
20-5-2	48,908	0.00	− 0.12
20-5-2b	37,542	0.00	0.00
50-5-1	90,111	0.00	0.00
50-5-1b	63,242	0.00	0.00
50-5-2	88,298	0.32	0.22
50-5-2b	67,308	0.21	0.22
100-5-1	274,814	0.56	0.67
100-5-1b	213,615	0.68	0.63
100-5-2	193,671	0.12	0.34
100-5-2b	157,095	0.04	0.35
100-5-3	200,079	0.21	0.28
100-5-3b	152,441	0.30	0.29
100-10-1	287,983	4.17	− 1.50
100-10-1b	231,763	3.68	− 2.03
100-10-2	243,590	0.80	0.71
100-10-2b	203,988	0.25	0.51
100-10-3	250,882	1.59	1.07
100-10-3b	204,317	0.91	0.93
200-10-1	477,248	1.25	1.65
200-10-1b	378,351	0.58	1.01
200-10-2	449,571	0.48	0.81
200-10-2b	374,330	0.48	0.71
200-10-3	469,433	2.12	2.17
200-10-3b	362,817	1.87	2.13
	Avg	0.79	0.42

H refers to the work of Hemmelmayr et al. (2012)

**Table 12:** Results for LRP on Tuzun instances

Instance	BKS	H (%)	ALNS (%)
111112	1467.68	0.54	0.42
111122	1449.2	1.07	1.21
111212	1394.8	0.41	0.49
111222	1432.29	0.62	0.98
112112	1167.16	0.50	0.44
112122	1102.24	0.01	0.38
112212	791.66	0.02	0.38
112222	728.3	0.00	0.32
113112	1238.24	0.17	0.36
113122	1245.31	0.23	0.19
113212	902.26	0.00	0.22
113222	1018.29	0.03	0.10
131112	1866.75	3.90	4.28
131122	1833.95	1.27	1.54
131212	1965.12	2.26	2.41
131222	1801.39	2.06	2.38
132112	1443.33	0.40	0.80
132122	1441.98	0.34	0.25
132212	1205.09	0.06	− 0.04
132222	930.99	0.23	0.63
133112	1699.92	0.03	0.05
133122	1400.01	0.25	0.25
133212	1199.51	− 0.02	0.11
133222	1152.18	0.19	0.40
121112	2259.87	0.81	0.86
121122	2185.41	0.33	0.61
121212	2234.78	0.58	0.84
121222	2241.04	0.99	1.02
122112	2089.77	0.19	0.52
122122	1709.56	1.31	1.34
122212	1466.62	− 0.30	− 0.23
122222	1084.78	0.12	0.17
123112	1970.44	0.03	0.29
123122	1918.93	1.74	1.95
123212	1771.06	− 0.39	− 0.05
123222	1393.16	0.16	0.23
	Avg	0.57	0.74

H refers to the work of Hemmelmayr et al. (2012)

## Footnotes

1 http://prolog.univie.ac.at/research/PDPRegions/PDPRegions.zip.
